# The Hospital. Nursing Section

**Published:** 1905-09-30

**Authors:** 


### The Hospital.

### Nursing Section.

Contributions for this Section of "The Hospital" should be addressed to the Editor, "The Hospital" Nursing Section, 28 & 29 Southampton Street, Strand, London, W.C.

No. 992.—Vol. XXXVIII. SATURDAY, SEPTEMBER 30, 1905.

### Notes on News from the Nursing World.

### AMERICAN NURSES AND PATRIOTISM.

The nursing topic of the hour in the United States appears to be the failure of the nurses to volunteer for service in the Army Nurse Corps. In March last year the superintendent of the Army Nurse Corps, Mrs. Kinney, issued, at the instance of the Surgeon-General, an official intimation that it was desired to obtain the names of acceptable graduate nurses willing to serve in time of war or national emergency. The requirements for enrolment were fully set forth, and attention was called to the announcement by the American nursing magazines. Mrs. Kinney subsequently issued a circular letter to the superintendents of approved training schools asking for their co-operation in securing a representative body of women for this department of the service, and the superintendents are said to have heartily endorsed the plan and rendered every assistance. Yet the practical result of these efforts is that up to the present time only 41 nurses out of over 30,000 have expressed their desire to serve their country in time of need. If this state of affairs is not likely to be improved by the hysterical language of a periodical which is moved to cry aloud, "How long, O Lord, how long?" it must be admitted that the situation is not promising, and that State Registration in America does not seem to further the development of patriotism.

### NURSES AND UNTIDINESS.

We frequently receive letters pointing out the untidy appearance which characterises many nurses wearing outdoor uniform. But a correspondent whose initials conceal a well-known personality affirms that she considers "that the failing most common to nurses is untidiness." This opinion is based upon her experience during two dangerous illnesses, and she thinks that this failing is the cause of more trouble, and consequent "ructions" in a house where sickness prevails, than anything else. Of course, her remarks apply only to private nurses, and we have no doubt that there are some who need the constant discipline of hospital life to make them pay due attention to neatness and order. But to the woman whose vocation is nursing, and who does not merely follow it for the purpose of earning money, untidiness in her general habits should be as impossible as vulgarity or an offensive manner.

### PROPOSED ALTERATION OF A SOUND RULE.

In the paper on "Town and Country Nursing," which was read by Dr. Henry Barnes at the Carlisle Diocesan Conference the other day, and is reported in another column, attention was called to a proposal on the part of certain district nursing associations in the country to delete the rule that the nurse shall only act in conjunction with the doctor, except in cases of grave emergency. In other words, each nurse employed by the associations, being registered under the Midwives Act, and therefore entitled to practise midwifery, it is suggested that she should be allowed to attend cases independently of the doctor. We think with Dr. Barnes that this would be a fatal mistake, and his remarks on the probable consequences of its perpetration may be commended to all who are inclined to favour a concession which, as he remarks, would almost certainly lead to unqualified practice in regard to minor ailments and accidents of all kinds. There is but a single safe course to pursue, and it is embodied in one of the six propositions with which Dr. Barnes closed his paper. If midwifery nursing is undertaken independently, it should be under well-defined conditions, and be the exclusive work of the nurse.

### ONLY A FEVER NURSE.

A correspondent who has lately been appointed matron of an isolation hospital in the Home counties, and was chosen from among 50 candidates for the post, complained last week that there is a disposition on the part of the modern three-years'-trained nurse to look down upon any nurse not so trained as necessarily incompetent. We fear that the complaint is not unfounded, and we entirely sympathise with the fever nurse who winces when she hears young women who have only just secured their three-years' certificate remark of nurses with four times their experience, "Oh, she is only a fever nurse." The competent fever nurse, whatever her lack of general training, is a most useful person, to be respected instead of looked down upon. We attach so much importance to the experience to be gained in well-conducted hospitals for infectious diseases, that we advise nurses who have finished their general training, instead of talking about "only a fever nurse," to learn for themselves the duties which she has to discharge.

### RESIGNATION OF THE MATRON OF THE LONDON HOMŒOPATHIC HOSPITAL.

Miss Brew, matron of the London Homœopathic Hospital, has soon followed the example of Miss Thorold. It is now more than 30 years since she left the Royal Southern Hospital in Liverpool to enter upon her duties in the unique London institution. At that time the Homœopathic Hospital was an extremely inconvenient building, and it was not until 20 years later that it was replaced by one more suited to the requirements of the day. Even as it is, the arrangements of the Nurses' Home leave something to be desired. Owing to restrictions of space, the nurses have to sleep in dormitories containing six beds, each of which is curtained off, instead of in separate bedrooms. But Miss Brew has all along done her utmost to promote the welfare of the nurses, as well as the interests of the hospital. Under her auspices the age of the admission of probationers has been raised to 22, but she has always refused to regard an age limit as a law of the Medes and Persians. One of the nurses attached to the institution entered it before she was 20, and another after she had turned 30. Miss Brew has, of course, usually adhered to the regulations, but she holds very strongly that the personality of a woman is more important than her age. There is a private staff attached to the Homœopathic Hospital, and all its members were trained in the school. In 1902 Miss Brew started a nurses' co-operation for the convenience of the medical men and with the view of retaining the nurses connected with the hospital. The private nurses make all the curtains, the sheets, and the quilts for the children's ward; and special attention is given to a ward devoted to girls between 17 and 24. Miss Brew, throughout her long period of office, has spared no pains to urge upon all associated with her the importance of maintaining in the hospital the atmosphere of home.

### MIDDLESEX HOSPITAL.

We learn, with much pleasure, that the resignation of Miss Thorald is to be marked by a presentation which will take place in November. For the present at all events there will not be any changes in the nursing department at the Middlesex Hospital. Miss Thorald is still at her post, and the whole of the nursing staff have now returned to duty.

### NO NURSING TOPICS AT THE WOMEN WORKERS' CONFERENCE.

It is rather strange that the programme of the Conference of the National Union of Women Workers, which, as we announced last week, will be held at Birmingham from the 23rd to the 27th of next month, does not include any papers on nursing subjects, nor does the name of a nurse appear in the list of published speakers. It is true that some of the questions to be treated at the Conference are more or less interesting to nurses, but in the circumstances it can hardly be expected that its proceedings will be followed by them with keen attention.

### THE MATRONSHIP OF THE NATIONAL HOSPITAL FOR THE PARALYSED.

This is the last week for receiving applications for the post of matron of the National Hospital for the Paralysed and Epileptic, Queen Square, Bloomsbury. We hear that several exceedingly eligible candidates have applied, and it is probable that the difficulty of the governing body will be to select the best. The duties of the post, however, require the appointment of a woman of marked capacity, whose experience is not confined to a single hospital in London.

### THE TESTIMONIAL TO MISS PETER.

It is hoped to present Miss Peter, the retiring superintendent of the Queen Victoria's Jubilee Institute, with a testimonial at the United Conference of Northern and Southern Superintendents of Nurses in November. This would be a very suitable occasion for the ceremony, and the arrangement would, we are sure, afford the maximum of pleasure to Miss Peter and to all concerned. The presentation, we hear, will be in the form of a cheque.

### ENTHUSIASM AND ROUTINE WORK.

At the third annual meeting of the Heswall District Nursing Association, an address was delivered by Miss Gillie, secretary of the Liverpool Queen Victoria's Nursing Association, who prefaced her remarks by congratulating the Association on their financial position. She confessed that she could not help feeling a little envious at their balance, because in Liverpool there was invariably a deficit. Miss Gillie subsequently sketched the history of district nursing and showed its development, particularly in Liverpool, the cradle of its birth, and she received a most cordial vote of thanks for her address, one of the speakers observing that, after hearing it, the nurses in Heswall would perform their duties with increased enthusiasm. That we can fully believe, and there is no doubt that the delivery of such addresses, in which the broader aspects of nursing are treated, are distinctly calculated to encourage and help those who are engaged in routine work.

### A NURSES' HOME IN MEMORY OF A DOCTOR.

An important presentation has just been made to the representative of the Ladies' Committee of the Holywood District Nursing Society. The home, erected to perpetuate the memory of Dr. Dunlop, which was started a short time after his death three years ago, has been completed, and the formal handing over of the lease and the key of the building was an event of interest to all who know how closely Dr. Dunlop had the welfare of the association at heart. The District Society has been in existence for twenty years, and it is now intended to make the new building the centre of its operations in the neighbourhood. It provides accommodation for the two nurses, and also beds for the reception of such emergency cases as can be treated there instead of sending them at once to the hospitals in Belfast. The house is built in the old English cottage style, and in addition to the rooms for the use of the nurses, there is a kitchen, a scullery, and a store for surgical appliances, two small wards for patients, and a bright, airy room for the children. The Ladies' Committee desire to form an endowment fund, towards which they have in hand £350. The house, moreover, is still in need of furniture and equipments. These additions, it is hoped, may be partly done by residents in the locality familiar with the good work of the society.

### MATERNITY NURSES AND CLEANLINESS.

The medical officer of the Littlehampton district has been compelled to call the attention of the East Preston guardians to a tendency on the part of the mothers to refuse the attendance of the professional maternity nurse, whose services are given gratuitously at the instance of the Littlehampton Nursing Association, on account of her insistence on conditions of cleanliness which they have not been in the habit of observing. He asked the guardians to relieve him of responsibility in such cases and enable him to cancel the order if the patient declined the services of the parish nurse and employed "ignorant and dirty women." As the result of a long discussion it was decided that the request should be further considered. We do not understand the hesitation of the East Preston guardians in supporting the medical officer in the course which he naturally proposes to take.

### FREQUENT RESIGNATIONS AT ATCHAM.

The Atcham Guardians are troubled by the frequent resignation of members of the nursing staff. At their last meeting it was signified that two nurses, one of whom had only just been appointed, desired to leave. The latter, in fact, was willing to forfeit her month's salary rather than remain. It was said that she wanted to go because she could not agree with the rest of the staff. No reason was suggested for the resignation of the other nurse. The Guardians decided to make an inquiry, and we think that it is necessary. A nurse does not often want to relinquish her post a few days after her appointment has been approved by the Local Government Board. The chairman is in favour of not giving a testimonial to a nurse who does not stay at the infirmary for twelve months, but this would hardly tend to stem the tide of dissatisfaction.

### DISTRICT NURSES AND POOR-LAW WORK.

The Machynlleth Guardians have decided to subscribe £5 to the District Nursing Association on condition that the nurse attends to patients inside the Workhouse. We assume that the consent of the committee of the Association has been obtained, but the arrangement is peculiar. It is not, we think, at all desirable that district nurses should undertake duties which belong to the workhouse staff. On the other hand, such an arrangement as that just made by the Glanford Brigg Guardians in Lincolnshire is entirely admirable. It having been reported to them by the medical officer that during the last six months the district nurse had paid 300 visits to the poor, the Guardians have agreed to subscribe regularly to the Kirton-in-Lindsey Nursing Association.

### AN ALLURING PICTURE OF PANAMA.

A good deal of indignation has been excited among the nurses at work in Panama by statements made in the New York papers that the conditions in the hospitals and the status of the nurses are very unsatisfactory. The nurses employed in Ancon Hospital have categorically denied the accuracy of these statements. Thus, they affirm that the home conditions provided for the nurses, instead of deteriorating, as insinuated, are steadily improving; that as to climatic disadvantages, there has only been one case of serious illness among the nurses since their arrival more than a year ago; that, instead of it being true, as alleged, that the only nurses who go to Panama are those who are tired of private duty and desire a change, there are many who left good hospital positions in the United States because the memory of previous work in the tropics was too strong to allow them to remain in a country less favoured in these respects. It is admitted that various little useful accessories are not always forthcoming, but among compensations for trifling drawbacks are said to be eight hours' duty; a week's rest at Tobozo, a beautiful island in the Pacific Ocean, at the completion of four months' service; six weeks' vacation at the end of eight months' work, with many interesting places in which to spend a vacation if a nurse does not wish to go home; and perfect liberty to come and go when off duty, untrammelled by rules and regulations.

### WEDDING PRESENT FROM OLD PATIENTS.

On Monday last, at Southsea, Miss Maude Rodgers, daughter of the late Inspector-General of Hospitals and Fleets, was married to Fleet Surgeon J. L. Smith, R.N. Until her marriage Miss Rodgers was assistant matron at the Parish of Portsmouth Infirmary, and amongst the large number of guests present at the wedding were Dr. Charles Knott, medical superintendent to the Infirmary, Mrs. Knott and a large number of nurses. The presents included a set of sugar bowls and spoons from the nurses of the Infirmary, and a gift from some of the old patients at St. Pancras Infirmary, where Miss Rodgers was at one time charge sister.

### NURSING AT PITLOCHRY.

The ninth report of the Irvine Memorial Nursing Home and District Nursing Association at Pitlochry was adopted at the annual meeting. During the year 1904 the Nursing Home was largely taken advantage of by the sick and suffering, who evidently fully appreciated its benefits. Six patients, instead of four, can now be admitted, a bed and a child's cot having been added to the ward. The number of patients during the year was 31, 13 of whom were admitted free. The Committee have for some time been considering the advisability of adding to the Home a room for general purposes and of increasing the accommodation for the staff. This it was resolved at the meeting should be done, provided that the needful funds are guaranteed. The Committee complimented the nurse-matron upon the reasonable amount of her housekeeping expenses, adding that the medical men considered her most efficient in the discharge of her duties, and the patients showed her much gratitude. As to the district visiting, 1,741 visits were paid to 79 patients, the greater proportion being gratis.

### THE CALIBRE OF ASSISTANT NURSES.

The question whether the standard of assistant nurses is deteriorating was raised at the last meeting of the St. Thomas Board of Guardians, Exeter. The guardian who propounded it supported his contention that the answer to the question is in the affirmative by pointing out that of five candidates who had been asked to attend before the Board only two had condescended to comply with the request. This, at any rate, was not polite, though it ensured the appointment of the two who were present to the two vacancies. We can understand, however, that the standard of assistant nurses in Poor-law institutions may be deteriorating because the need of full training is so generally recognised that young women do not care, as a rule, to enter upon a nursing career through a sort of back door, which cannot, in ordinary circumstances, lead to promotion.

### The Nursing Outlook.

"From magnanimity, all fear above;
From nobler recompense, above applause,
Which owes to man's short outlook all its charm."

### CENTRAL EXAMINATIONS IN AUSTRALASIA.

MOST of the mistakes made by the advanced advocates of State Registration in this country have been due to the failure to recognise the factors in the problem presented by the actual state of nursing in the United Kingdom compared with that in other communities and nations. In the United Kingdom for practical purposes, the only real training available for nurses is confined to those institutions where there is a properly organised training school. These training schools are practically responsible for the training of every British nurse who has attained an adequate standard. There are no doubt a large number of women engaged in nursing who have never been properly trained at all, and each year their number is added to by the product of small special and cottage hospitals which have neither the requisite number of beds, nor the facilities to train nurses, and by the output of the fever hospitals where no general training is possible at all. Provided, therefore, the organisation of nursing by counties under some such scheme as we recently formulated can be carried out, the establishment of a uniform curriculum and the enforcement of a uniform standard of efficiency for all probationer nurses should prove easy of accomplishment.

In Australasia, as we have already explained, the hospitals are subsidised by the Government, and are so under Government control that Registration has been easily enforced, but even there the so-called training of nurses is undertaken by large and small hospitals each of which grant certificates of training. The Australasian Trained Nurses' Association endeavour to meet the case by enacting that candidates for Registration must produce proofs that they have been engaged (1) for three years in a general hospital recognised by the Council, having an average of not less than forty beds occupied daily, or (2) that they have been engaged for four years in a country district or suburban hospital recognised by the Council, with an average of twenty beds occupied daily, or (3) for five years in a private country district or suburban hospital recognised by the Council, with an average of not less than ten beds occupied daily. This recognition of small cottage and private hospitals as training schools has created a difficulty with the Victoria Trained Nurses' Association, and has created marked differences in the quality of the nurses and the qualifications they possess on becoming registered.

With a view to remove these difficulties, at the annual meeting of the Australasian Association, recently held, it was unanimously decided that on and after June 1st, 1906, no nurse trained at a training school, general or special, recognised by the Australasian Association should be admitted to membership until she had passed the final examination. This examination is to be both written and practical, and to comprise all subjects in the regular schedule except anatomy and physiology. No nurse will be eligible for the final examination until she has completed the required course of training, and each candidate must produce a certificate of having attended lectures and passed a satisfactory examination in physiology and anatomy at a recognised training school, and also a certificate of good conduct. The examination papers will be set by a conjoint board of examiners, consisting of the boards of each Branch, and these papers will be sent under seal to the various centres. After each examination the candidates' papers will be sealed and sent to the examining board. Centres will be appointed in New South Wales, Queensland and South Australia, West Australia and Tasmania. An entrance fee of one guinea will be charged for this final examination, such fee in the event of the candidate passing will entitle her to full membership of the Association free for the first year, and in the event of failure, a nurse may enter for succeeding examinations without extra fee. A further step in advance was made by the Association which has determined that after the 1st of June, 1906, an educational test shall be applied to each candidate before admission to the final examination, and the production of proof of having attained to the standard of education laid down by the Council will have to be produced by every candidate. Under the educational test every intending probationer will apply in the first instance to the matron of a recognised hospital, who will refer the application to a Committee known as the Educational Committee for each State. This Committee will accept certificates that the candidates have passed a university examination or the sixth form standard of a primary public school, but all other candidates before commencing their training must pass an examination in general education under the supervision of the Educational Committee.

The proposals of the Australasian Association show the thoroughness which characterises the action of the Colonial authorities in matters affecting the general welfare of the whole community. The institution of a central examination in the special circumstances which attach to Australasia is undoubtedly a sensible method of benefiting the higher education of nurses up to a definite standard, and thus improve the quality of the nursing service everywhere. The same result can be attained by different means with equal efficiency in the United Kingdom, but the action of the Australasian Association and its results cannot fail to be watched with deep interest by nursing authorities all the world over.

### Medical Electricity and Light Treatment.

By Kate Neale, Sister-in-Charge of the Actino-Therapeutic Department, Guy's Hospital.

X.—FINSEN LIGHT (*continued from p. 400*).

### Treatment.

Treatment by light is apt to be a tedious and irksome business and one needing the exercise of much patience and care. In none of the methods described in earlier chapters does success depend so largely on the nurse and the conscientiousness with which she sets about her task. Inattention even to a trivial detail may mean complete failure with consequent loss of time, labour and expense. You may at first find the method of treatment not easy to acquire and the muscular fatigue it entails a temptation to relax in some measure the close attention you should spend on your work. Remember, however, that with a little practice difficulties will vanish and your muscles become inured to what was at first an unwonted strain.

In giving you a practical account of the light treatment I shall group my remarks under the following headings:—

I. Preparation of Patient.
II. Preparation of Lamp.
III. Application of Treatment.
IV. After-Treatment.
V. Special Practical Points.

I. **Preparation of Patient.**—By far the greatest proportion of cases that come for treatment exhibit the disease already advanced to ulceration, and in many you will find the raw surfaces crusted with scabs and dirt, the result of long neglect. Light treatment is of no avail while the skin is in this condition, and your first care must be to make the ulcers clean. Hot boracic fomentations, or special ointment must be applied frequently until the scabs are softened enough to be removed. Explain to the patient the necessity of keeping the lupus patches absolutely clean, and of applying the dressings regularly. When at last the skin is got into proper condition treatment may commence. If there is any ulceration of the skin a preliminary course of *x*-rays is usually ordered, because an ulcer is too tender to allow the application of the pressure-lens, and until the ulcer is healed the light cannot be used.

Before getting the patient ready the doctor or sister will have drawn a circle with a blue pencil—especially made to write on the skin—round the area you have been instructed to treat. This will serve as a guide during treatment to inform you if the pressure-lens has slipped. Let the patient lie on a narrow couch of such a height that he will be brought within a few inches of the lower end of the telescope. Make him comfortable with pillows, some large and others small for the nape of the neck, etc. He will have to remain absolutely still during the whole treatment, so a little time in arranging him is well employed.

II. **Preparation of Lamp.**—The water circulating through the telescopes must first be turned on, and not until you actually hear it flowing is the treatment to be started. If this precaution be omitted the heat of the lamp will, in a very few seconds, crack the lenses, and damage to the extent of many pounds will be wrought. It is not sufficient to turn the water tap: the actual flow of the water must be ascertained either by listening or by feeling the current in the india-rubber tube. I remember an occasion on which the tap was turned and the light switched on, but, unknown to the nurses, the water had been cut off at the main, and in a very short while a costly lens had cracked. The carbon points of the lamp may need attention, but this duty devolves on the electrician in attendance.

It is advisable to hang from the metal cylinder surrounding the carbons a flounce of some red material—such as soft muslin, or Turkey twill, doubled, and about twenty inches deep—to screen the glare of the light from neighbouring eyes.

Your next care must be to make certain that the telescope is pointing direct at the light. To do this remove the cap from its lower end and catch the beam of light on a sheet of white paper. A circle of bright light will appear on the paper. If on any side of this circle a shadow appears you will know the telescope lies out of the true, and needs deflecting *in the direction of the shadow*. On the body of the telescope you will see screws, by moving one or other of which, proper adjustment can be made until the shadow has faded. Replace the cap.

The pressure-lens now needs attention. Connect it to the two india-rubber tubes provided for the purpose, and turn on the flow of water. If any air-bubble is noticed inside it must be got rid of before proceeding. This can be easily done by turning the lens so that the rubber tubes are above when the bubble will rise to the tubes and be swept away by the current of water. Its disappearance can often be assisted by nipping one of the tubes and then suddenly releasing it.

All this preparation, including the adjustment of the telescope, must be repeated with each fresh patient.

III. **Application of Treatment.**—When you come to apply the light you will find that one of the great difficulties met with is to focus it accurately on the patient, and only by constant care during the progress of treatment can success be ensured.

Move the couch on which the patient lies so as to bring the area marked out approximately in focus—that is to say about four or four and a half inches from the end of the telescope. Still keeping the cap on the telescope, you will see on the patient's skin a circle of light with scolloped edges suggesting the outline of a flower, and sometimes called the "floral design." This light must fall exactly in the circle enclosed by the blue mark, and its outline must take as nearly as possible the form of a circle.

If the floral design be too large—it should be the size of a sixpence—move the patient further away from the telescope; if too small approach him nearer. The last and final adjustments should be effected not by moving the couch, but by the arrangement of small pillows behind the patient.

You are now ready to begin the treatment. Seat yourself on a stool high enough to enable you to look down on the patient, and having assumed your dark spectacles take hold of the pressure-lens so that its four handles point upwards, and grasp each of two opposite ones between finger and thumb. Then gently place it against the skin marked out, and steadily press firmer and closer until every trace of blood is driven out of the part. Now glance at the perforated metal cap again. You ought to see in its centre a shadow reflected up from the pressure-lens. Often it will not be quite in the centre, in which case you must alter the angle of the pressure-lens, without, however, releasing the firmness of your pressure, until you have brought the shadow to the exact centre of the cap. Lastly, with one hand temporarily taken from the pressure-lens, remove the cap. The full brilliancy of the light falls on the skin and the treatment has begun.

You will readily see that the two chief difficulties you have to contend with during the actual treatment are—firstly, the continuous maintenance of pressure necessary to keep the part bloodless; and secondly, the tendency of the pressure-lens to move ever so little and so allow the reflected light to wander from the centre of the lowest telescope lens. The former difficulty you will find not great after a little practice, but when you first begin your fingers get cramped and your arms ache long before the treatment is over. Gradually, however, this strain on your muscles becomes less noticeable, till after a time you can use the lens for an hour before feeling any discomfort. As regards the movement of the lens you will soon learn how to recognise any such change. For instance, you should every now and again hold your open hand on this side or that of the end of the telescope. If the lens has moved to any extent you will catch the reflected light on your palm and so be informed how the pressure-lens must be altered. With a little practice you will detect the error even more easily, and in this way: the telescope tube is prolonged for about one-third of an inch beyond the lowest lens, and the inside of this projection is usually in shadow but as the reflected light begins to creep away from the centre of the lens it will soon reach this ring and lighten it up on one side, and so reveal the movement.

Once again, let me emphasise the fact that the success of the treatment depends on the conscientiousness with which you attend to these details.

One hour is the usual duration of a single treatment. At the end of that time cover the end of the telescope with its cap, and remove the pressure-lens. Do this, however, gradually. Have you ever noticed after standing for some time on one foot, transmitting your weight through the one heel, how, when you move, the blood rushing back to the heel, pains? It is the same, but worse, after using the pressure-lens; therefore release the pressure by degrees.

(*To be concluded.*)

### The Nurses' Clinic.

### FEEDING WITH THE ŒSOPHAGEAL TUBE.

THIS form of feeding is chiefly used in diseases of the œsophagus. The difficulty in these cases cannot be met by nasal feeding, for the question is not how to get the food into the patient's throat, but how it is to pass into the stomach. The tube has to be passed into the patient's stomach and the food poured down it. As about 14 inches of thick rubber tubing has to be swallowed, this is naturally a more troublesome matter than nasal feeding, and can hardly be done without the patient's co-operation. The requirements for this mode of feeding are much the same as for feeding through the nose, except that the œsophageal tube is substituted for the rubber catheter, and a further length of tubing, of the same calibre as the feeding tube, is joined to it by means of a small glass tube about 3 inches long, the neck of the funnel being inserted into the other end. The nurse then must have ready:—

1. The feeding tube, comprising the œsophageal tube (or stomach tube, as it is often called), about 3 feet of rubber tubing of the same size and the small piece of glass tubing connecting the two.
2. A large glass funnel to fit the tube.
3. A large bowl of boric lotion, warm.
4. Three receivers: one for the funnel and tube, and two for use in case of vomiting, which may be copious.
5. Glycerine to lubricate the tube. Vaseline is unsuitable here.
6. A piece of mackintosh and several towels to protect both patient and bed.
7. The food, warmed, and in a glass pint measure; also any stimulant or medicine ordered to be given with or after it.

For feeding in this way the patient should be in a sitting posture, and, if in bed, should be well supported by pillows, a towel and mackintosh being arranged over the bedclothes. If, however, the patient is up and about, he should sit on a chair with a mackintosh under and in front of it, and another, with a towel over it, should be placed across his knees, a towel should be pinned round his neck, like a bib, and a large receiver be given him to hold.

The patient, especially when the feeding-tube is passed for the first time, is very likely to vomit. It is most important in these cases that the nurse should gain her patient's confidence, neither hurrying nor exciting him, but going about her work in a confident and quiet manner. She must reassure the patient by telling him that after the first time the passage of the tube is an easy matter, and that he will soon be able to do it for himself without help. Often this mode of feeding has to be employed for months, or even years. The nurse should have assistance on the first few occasions, or until the patient is able to pass the tube for himself. A piece of white silk or cotton should be tied tightly round the tube, of course not tightly enough to compress it, 16 inches from the eye. This cotton marks the distance from the teeth to the cardiac orifice of the stomach into which the tube passes. If passed further it may injure the coat of the stomach, therefore the cotton should not be allowed to pass the teeth as a rule.

All being ready, the patient should incline his head a little forward rather than backward, and the nurse should put her left forefinger into his mouth, as far back as she can, holding down the tongue gently and firmly. She should hold the tube between the forefinger and thumb of the right hand, passing it over the left forefinger, till it reaches the back of the throat, when she should tell the patient to swallow and pass the tube forward as he does so. Should it be a case of stricture, the tube will probably pass fairly easily till the narrowing is reached, when firmer pressure will be needed to get the tube past it. Force, however, must on no account be used, and the tube must be withdrawn and the fact reported, if, with ordinary firm pressure, it cannot

THE NURSES' CLINIC—*Continued.*

be passed. Any vestige of blood on the tube must of course be reported also. A good deal of retching and vomiting may accompany the passage of the tube, but it is not necessary always to withdraw it if the patient can be persuaded to retain it. When the stomach is reached, gas will probably come up through the tube. This should be allowed to escape before the funnel is filled with the food. When once filled it should be kept full. The assistant nurse should be responsible for this; if the funnel be allowed to get empty and then be filled up, air will be forced down the tube into the stomach and cause distress and perhaps further vomiting. If the feeding has to be interrupted for cough, or any other reason, the tube should be tightly pinched between the finger and thumb, and so held until the feeding can be resumed, the funnel being filled up and any air bubbles allowed to escape before the pressure is relaxed. Sometimes the funnel remains full and the fluid will not pass down the tube, very often it is because the eye is pressed upon by the stomach wall and withdrawing the tube an inch or two or letting it go a little further in may put matters right. Sometimes air in the tube is the cause, or the tube has become blocked by a curd of milk; if the patient is asked to cough, the impulse from the stomach may clear the way. As a last resource it must be withdrawn and passed again, after having been well washed, but this is very hard on the patient, so the nurse must take every care to avoid the necessity. The food should always be strained, and any medicine ordered, unless there are directions to the contrary, should be given towards the end, as the funnel is emptying. The tube and funnel must of course be boiled between each meal and kept in boric lotion covered up.

In some cases of disease, or obstruction of the œsophagus, the stomach tube cannot be passed, owing to the risk of rupturing an aneurysm, which may be the cause of the obstruction, or, where there is a malignant growth, of making a false passage into the trachea, or anywhere through the diseased wall of the œsophagus. The operation of gastrostomy is performed in such cases, an opening (Latin, *os* a mouth) being made into the stomach from outside and a tube passed into it through the opening, by means of which the food is given. In some cases the tube, a rubber catheter, has to be passed into the stomach each time the patient is fed, and the nurse must be very careful when doing this that the tube does go direct into the stomach; a case has been known where a false passage was made and the food found its way among the tissues.

In other cases the tube is left in situ and is only taken out and replaced by a clean one when the wound is dressed. All the nurse has to do here is to insert the neck of a glass funnel into the catheter and pour the measured food through it, wrapping the end of the catheter in a piece of rubber tissue, to prevent leakage when the feed is finished, and replacing the covering wool and bandage. After a time the patient is able to feed himself through the tube and continues to do so as long as he lives.

### Town and Country Nursing.

At the Carlisle Diocesan Conference last week Dr. Henry Barnes read a paper on "Town and Country Nursing" which excited general interest. After tracing the history of nursing back to the Crimean War—in the course of which he paid a glowing tribute to Florence Nightingale and her nurses—he alluded to the school for nurses established at St. Thomas's Hospital, and to the work of Mr. William Rathbone in Liverpool, which led to the establishment of Queen Victoria Jubilee Institute, and to the institution of county nurses. Having also described the difference which exists in the training of these two classes of nurses Dr. Barnes continued: During the last few weeks I have made many inquiries from professional friends and others as to how far the nursing of the sick poor is provided for in this diocese, whether any suggestions can be made for its improvement, and whether any special difficulties have been encountered.

### Nursing in the City of Carlisle.

In the city of Carlisle, district nursing was started in 1891, and was at first confined to the Denton Holme district; it speedily extended to other parts of the city, and four years ago, the Carlisle District Nursing Association became affiliated with the Queen Victoria Jubilee Institute. It has one superintendent and three nurses, all Queen's nurses. Last year these nurses paid over 13,000 visits, and the inspector of the Jubilee Institute reports the work as "very satisfactory." have had knowledge of the work since it started, and I thoroughly endorse this opinion. The work of the Carlisle Association is increasing year by year and financially its position is prosperous. It nurses the sick poor free of charge, but patients are encouraged, when able, to give small donations, and about £25 was obtained in this way last year. There is in Caldewgate, Carlisle, a separate nursing organisation employing one nurse, who is stated in the last report I have been able to obtain, to have made 8,737 visits in one year. She is not a Queen's nurse. The Carlisle Maternity Association was started in 1895. It employs one nurse, who attended 163 cases last year. It is supported partly by subscriptions, but partly also by fees, £47 18s. 6d. being obtained from the latter source in 1904. In these two societies there is no systematic inspection of the work of the nurses, and it would, I think, conduce to greater economy in management and greater efficiency if all the societies doing district nursing in Carlisle were amalgamated. Whitehaven and Workington have separate district nursing organisations, the first-named being affiliated with the Queen Victoria Jubilee Institute. Whitehaven also has a maternity charity. There is a nursing association for Farlam and Midgeholme, mainly a colliery district. It was started by Mrs. Lacy Thompson in the Coronation year, and has been highly successful.

### The Cumberland Nursing Association.

Under the auspices of the Countess of Lonsdale, the Cumberland Nursing Association was established in 1897, its aims being to bring the benefits of skilled nursing within the reach of all classes, both in town and country. It has formed districts in various parts of the county, each district being under the management of a local committee. It has selected nurses for training and afterwards appointed them to districts. It has a superintendent who is a Queen's nurse, and she periodically inspects the work of all district nurses affiliated with the County Association. The County Council makes grants in aid of training suitable women for nurses, and the training of others is provided for by a separate fund. According to the last report of the Cumberland Nursing Association, there are five districts employing Queen's nurses—namely, Maryport, Cleator and Cleator Moor, Millom, Cockermouth and Papcastle, and Longtown. There are 31 village nurses working under the supervision of the County Association. The nurses do not attend infectious cases. As a rule they attend midwifery cases only in an emergency or where there is a doctor in attendance and requests their services. There is no provision for supplying emergency nurses. In the event of the nurse needing a holiday or having a sudden breakdown in health, there is no one to supply her place. The Secretary, Lady Mabel Howard, tells me that this is one of their most pressing needs. There are several areas in the county not provided with district nurses for lack of funds. It has been intimated to me that district nurses are greatly needed, and would be much appreciated in Silloth, Abbey Town, Harrington, Arlecdon and Frizington, and in several Fell side districts.

### Proposed Alteration in the Rules.

The work of the nurses is generally acknowledged to be very satisfactory, and beyond the difficulty of providing an adequate income for the maintenance of the nurses no difficulty of any importance has arisen until recently, when it was proposed to delete one of the fundamental rules of the district associations which provides that the nurse shall only act in conjunction with the doctor except in cases of grave emergency. By the passing of the Midwives Act of 1902, which came into force on April 1 of this year, nurses holding a recognised diploma are entitled to practise midwifery, and as all the nurses working under the County Association possess this qualification, it has been proposed to delete the above-mentioned rule, and allow the nurses to attend cases independently of the doctor. This would be a fatal mistake. It would inevitably lead to friction, as the nurses would be competing with the doctors for an important part of medical practice, and if backed up by strong local committees, the nurses would, in most instances, get the best of it. The fees accruing for this class of practice might go to augment the salary of the nurses or they might be counted as part of the ordinary income of the local charity, but in either case it would then cease to be a charity and become a business enterprise carried on for profit and subsidised by charity. If the principle were once conceded it is felt that there would be no stopping place, and that it would almost certainly lead to unqualified practice in regard to minor ailments and accidents of all kinds. In my judgment, the rule is a good one; it has worked well, and I think it should be continued. There is abundant work for nurses to do under existing regulations without undertaking fresh responsibilities which would occasionally severely tax the time and energies of the nurses who are, I believe, generally opposed to any alteration of the rule. If midwifery is undertaken independently of the doctor it should be by nurses who do no other kind of work, and there is plenty of scope for their undertaking such work.

### Financing the District Nursing Associations.

I have been much struck with the different methods adopted for financing the local district nursing associations. Some three or four of them are worked on the principle of no fees, the nurse being provided at the expense of a few benevolent friends in the district, but a large majority of them are carried on to some extent on the provident principle. As people value most what they pay for, it is, I think, advisable that all schemes for providing district nurses should, as far as possible, be placed on a provident basis, graduating the scale of payment according to the earnings or position in life, the labourer paying a smaller sum than the small tradespeople and farmers. This is much better than depending on a few generous donors, as, should their contributions be withdrawn, there is a risk of collapse. Further, the provident principle would have the effect of increasing the independence and self-reliance of those to whom the nurse is sent, instead of encouraging them to depend on the gratuitous help of the benevolent. Those who subscribe have the first claim on the nurse, but her services are freely rendered to all. Boards of Guardians, as a rule, willingly subscribe in order to enable the nurse to go to those very poor who are not able to contribute at all. In some places, the working men combine to support district nurses, and Stockton-on-Tees claims to be the first town where this has been systematically done. The system pursued is to deduct ½d. per week from the wages of all the men in the large works. In this way £388 was obtained last year, being more than £100 in excess of the subscriptions derived from other sources. In Carlisle the working men make substantial grants from the Hospital Saturday Fund in aid of district nursing. Many of the local associations in the county are well supported by the working classes, about one-third of their income in some instances being derived from small subscriptions of 5s. and under. At Cleator and Cleator Moor, however, the committee report that they have long recognised a potential source of income in workmen's subscriptions. Last year, out of an income of £215, nearly £100 was obtained from workmen employed at the various works in the district, and upwards of £20 in subscriptions of 5s. and under. This is an example well worthy of being followed.

### Need for Church Offertories.

Considering the importance and value of the work I have been surprised to see that church offertories are not more frequently given, and I hope that the attention that the subject will receive to-day will remove this reproach. It is a reproach which is keenly felt in some districts. As regards district nursing in Westmorland, there does not appear to be any county association on the same lines as the Cumberland Nursing Association, but I have reports of district associations at Kendal, Kirkby Lonsdale, Ambleside, Windermere, and Appleby. In the Furness district, the Barrow District Nursing Association employs three nurses, all Queen's Nurses, and the result of the inspection of their work from headquarters is recorded as "very satisfactory indeed." They have a Queen's Nurse in Ulverston, and Village Nurses at Haverthwaite, and at Cark and Holker. I have examined the reports from all these districts, and they merely confirm the views I have already expressed. There are many districts both in Westmorland and Furness still unprovided with district nurses, and I have been informed that there is urgent need for one at Dalton-in-Furness.

### General Propositions.

As the primary object of this paper is to elicit discussion, I venture to submit the following propositions:—

1. That local associations for providing nurses for the sick poor in their own homes are doing a good work and deserve support.

2. That such associations should be affiliated with the County Nursing Association or the Victoria Jubilee Institute, as they thereby secure inspection of the work of the nurses and raise the standard of nursing.

3. That Queen's Nurses be employed in preference to Village Nurses, if adequate means can be provided for their maintenance.

4. That local associations should be organised and conducted, as far as possible, on a provident basis.

5. That District Nurses should not attend infectious cases, nor undertake midwifery on their own account, except in cases of grave emergency.

6. That if midwifery nursing is undertaken independently, it should be under well-defined conditions, and be the exclusive work of the nurse.

In conclusion, I should like to quote two remarks as to the value of nursing the poor in their own homes. The first is by Mr. Charles Booth. In his book entitled "Notes on Social Influences," he writes: "Of all the forms that charity takes, there is hardly one that is so directly successful as district nursing. It is almost true to say that wherever a nurse enters, the standard of life is raised." The second remark was recently made to me by a resident in a neighbouring town where district nursing was started rather more than a year ago. In reply to my inquiry as to its working, he said: "It is generally agreed that the best thing that has ever happened to the town has been the arrival of the district nurse."

### Nurses on Holiday.

### IN THE SCHWARZA VALLEY, THURINGIA, GERMANY. BY A GUY'S NURSE.

Being entitled to six weeks' holiday, I decided to spend it abroad, choosing Germany, as I had never been there before. I had the desire also to become acquainted with the language and to pick up as much as I could of the ordinary everyday expressions in that short time. For this reason I went alone and lived among the German people.

Some years previously my brother had attended the Evangelical Alliance which is held annually at Blankenburg, in Thuringia, during parts of August and September; and knowing how much he had enjoyed the services, the company, and the beautiful walks in the neighbourhood, I fixed on that spot for my head-quarters. I thought I should learn the language best by meeting a large number of people. I wrote to Fräulein Nordsieck, who is at the head of the "Allianzhaus," inquiring the terms, and found that they would suit my requirements.

Having settled where to go, I had next to find out how to get there. Messrs. Thomas Cook and Son were able to help, and after looking at various routes I decided to go *viâ* Harwich and Hook of Holland and then by train to Gotha, changing at Düsseldorf and Hagen. I took second-class return tickets to Gotha and first return on boat, being about £4 8s. 5d.

I left Liverpool Street Station by 8.30 p.m. continental express, reaching Harwich about ten. We had a very good, smooth crossing, arriving at the Hook about five in the morning. A porter took my handbag and put me into the train for Düsseldorf. At Cranenburg we all had to get out and have our luggage examined, it being a frontier station. The country passed through was very flat, with nothing of any special interest to note.

Our first change was at Düsseldorf and then on to Hagen, where I had some time to wait. From Hagen to Gotha I went direct. My companions generally were agreeable, but I could hardly keep up any conversation, not knowing enough German.

It was a stifling hot day, becoming hotter as the day wore on. The country we passed through became prettier and more interesting, with wooded hills and bubbling streams.

At Cassel we had a thunderstorm, with torrents of rain, which cooled the air. We had a few minutes to wait, when we were glad to get out for a stretch of limbs and some refreshments. We now wound in and out among the beautiful hills, on one of which stood the fine old Castle of the Wartburg. By this time the storm had become very severe and the lightning gleamed on either side and through our carriage. It was very grand to watch.

At last, about 8.30, we arrived at Gotha, when I was very glad to get out. Here I found the porter from "Hôtel Wunscher" waiting for me, as I had taken a bedroom there. We had just got in when the rain came down in a deluge. I had a splendid large room for two marks, on the second floor. The next morning I found my way to the Castle of the Dukes of Saxe-Coburg-Gotha, when the custodian took me all over it. It seemed strange to see so many pictures of our Royal Family hanging on the walls. After seeing the Castle, for which they charge 50 pfennig, I went through the beautiful Park to see the Museum. In the afternoon I took the hotel omnibus to the station. There I had a little difficulty over my ticket, as I could not book direct to Blankenburg. I fortunately remembered the names of the stations, "Arnstadt" and "Neudietendorf," which were the two places where I had to change trains. At Neudietendorf the guard of the Arnstadt train said he was going direct to Blankenburg, so I should not need to change at Arnstadt, at which station he kindly renewed my two tickets. He also put me into a first-class compartment. This last part of my journey was the prettiest, the hills looking lovely after the heavy rain and the evening sun shining on the glistening leaves. By 8.30 p.m. I was delighted to see Blankenburg, where there was a kind lady to meet me. It all seemed so very strange at first, for there were about 20 visitors or more, ladies and gentlemen, but it was like one large family. I found them all most kind, and anxious to help me with the language. They told me it was a very difficult language—which I found it to be.

The little town is situated at the beginning of the Schwarza Valley, and surrounded by hills covered with beautiful pines and oaks. The walks through these woods are lovely, as are also the delicious smell of the pines on a hot day and the distant views to be had. On the ground were quantities of wild strawberries and bilberries, also wild flowers. The Verra and Schwarza are two beautiful rivers winding through this valley. I found this place such a complete change and rest, especially as I had spent eleven months of the year in the sick room. I noticed that the signposts along the paths and roads give the name of the place and the time it takes to get there, not the distance. Also, if there are two or three paths to different places, each place has a colour, which is painted on the trees here and there all the way along, so that one ought not to get lost!

It was curious to see the milch kine used for drawing carts, or for ploughing, and when the time came they were taken into their stalls and milked and fed. The peasants are very civil, saying "Guten Tag" or "Guten Abend" when you pass. The women and children go about bare-headed, and the latter bare-footed as well. The women do a great deal of heavy work in the fields, and carry heavy loads on their backs in the funny baskets made to fit the back, with a strap across the forehead and other straps round the arms. Sometimes they carry hay, wood, or even coal. The mothers have rather a good way of carrying babies. They have a long piece of material of full width, which is plain one end and very full the other, being gathered into a neckband on one side and bordered with a frill on the other, so forming a cape. The plain end is tucked round the child, which they carry in their arms and fling the rest round their shoulders, the other being the cape. These look so quaint and pretty, being made of some pretty print. Most of the perambulators have little curtains attached to the hood—a plan that might very well be adopted in England.

Blankenburg is very central for the many places of interest in Thuringia. It is full of associations in connection with the Great Reformation.

One day a party of us took an early train to Eisenach. From the station we got a tram to the Wartburg, that famous stronghold and Castle where Martin Luther was imprisoned by a friend just to keep him from really being taken prisoner by his enemies. We were shown his room, also the stain on the wall, where he threw the ink-pot at the Devil! From the Castle there is a grand panorama of the surrounding country. We walked from the Castle through the woods up winding paths to the Hohe Sonne where we had a beautiful view of the Wartburg in the distance. Then we went down again, through lovely woods with the sun casting such pretty shadows, down to the Drachen-Schlucht-a deliciously cool cutting in a cleft of a high rock, the sides all covered with moss and dripping with water.

We returned to the town by tram from Marienbad. The town is very old and quaint. In the market-place is a fine statue of Martin Luther. We also visited his house and saw the bed- and sitting-rooms he lived in. They are both so very small. By this time it was necessary to catch our return train, but we found we had just missed it, so we had to stay at Eisenach for the night. We put up at the "Christliches Hospiz," an institution carried on on the same plans as the "Allianzhaus." But this building is new and quite modern, with good water-supply and electric light. Herr Tietz and his wife are kind hosts. The next day we spent visiting other places of interest, returning to Blankenburg in the evening.

But it was now time for me to return to England, having had a most delightful time at the "Allianzhaus" with those kind-hearted Germans, among whom I hope I have made some lasting friendships, and whom I may some day meet again.

From Blankenburg I took the train to Erfurt, where I saw the Cathedral, in which there is the wonderful fresco of "St. Christopher carrying the Christ Child across the River." I went up into the tower and stood under the large bell. It is a pretty old town, some parts reminding me of Venice. I saw the outside of the monastery but was unable to go in. In the evening I went on to Weimar, where I had taken a room at the "Erbprinz Hotel." After tea I went out and looked for Schiller's house, which, after a little while, I found. It is a quaint little house and still kept up as he left it. Not far from his house is the beautiful statue of "Goethe and Schiller" standing together. In the evening I walked about the Park and saw Goethe's house and the garden behind. It is all so quaint.

The next morning I visited the Goethe Museum, which is full of many wonderful things and curios, also letters from all the foreign celebrities, including members of our Royal Family. In the room where he died is the famous picture "Mehr Licht," being his death-bed scene. The expression on his face is almost too true to life. In the Park I also saw the beautiful statue of Shakespeare. Weimar itself is a fine town, so clean and well-planned, and there are many more places of interest to be visited if only there had been time to do so. In the afternoon I went on to Cassel, where I was met by one of the ladies with whom I had become acquainted at the "Allianzhaus." There was much to interest one in this quaint old town, with its curiously built old houses, especially one corner where the sharp-angled windows overlapped one another, the top floor being well over the street. I saw the house where the brothers Grimm wrote their famous "Fairy Tales." I spent the evening with my friends.

On the next morning I strolled about by myself, first going to the "Residential Palace," which is a fine place, and, as the floors are highly polished, visitors had to put on huge felt boots, afterwards I saw one of the picture galleries in which were some fine pictures. Then I walked back through the Park to my friend's flat. After dinner some of us went by train to Wilhelmshohe, the Kaiser's favourite country seat, where he and the Kaiserin often stay. It is a beautiful place, especially the park. The chief attraction here is the artificial falls, where the water comes rushing down over rocks and ferns, ending in a Roman aqueduct, from which the water comes down with such force into a lake as to send up a cascade into the air almost 100 feet high. I was there on a Wednesday, but Sunday is the more important day, for then more water is turned on.

Before leaving Cassel the next morning I visited the market, and was very disappointed not to see any peasants in the national costume. I then came home by a slightly different route, feeling thoroughly rested and refreshed by my holiday. It would have been still more enjoyable with a companion.

### Among the Out-Patients in an Eye Hospital.

BY AN OPHTHALMIC NURSE.

THE routine work of sight-testing is too varied ever to become monotonous, but a tremendous amount of patience is necessary to arrive at good results. It is interesting to look at the different types of patients as they sit in the waiting-room talking softly with their neighbours about their various complaints. Here there may be a fat, comfortable-looking country woman, who has come with a big basket for a day's shopping, and has thought that she would take the opportunity of getting "measured" for a pair of spectacles, because she can't see to thread her needle as well as she has done. She is a real trial to test, and will tell you confidently that she can see "all they letters"; but it is a work of art to make her read them properly, and harder still to choose the right spectacle for her, for to each lens that is put in front of her eye she says placidly, "That's better."

Worst of all to test are the small children from orphanages. I remember one little girl who had evidently been taught very good manners. To every letter that I pointed she would whisper, "The letter E" or "The letter M," at the same time, bobbing up and down on her chair, in place of the curtsey which she would have given had she been standing. I tried to persuade her that she need not be so polite and that I would greatly prefer that she should read the letters as they came without any preamble, but it was of no use, so I had to be resigned and get the best results that I could.

Once I came across a poor old shoemaker who was very much troubled by some filmy spots which danced in front of his eyes. He told me that he had pulled out lots of his eye-lashes thinking that they were the cause of his trouble, and that the spots looked like a fly with long legs. On testing his vision I found that he could see the letters at a distance fairly well, but that reading was almost impossible for him, because, as he said in his country dialect, "The legs of thic thur vly do get in the way of the letters."

I was very sorry for the poor old fellow, for the doctors were not able to help him very much, and I am afraid that he would have the company of that fly for many a long day.

It is most interesting and satisfactory when one can find the correct glasses for a patient who has some months before come to the hospital quite blind with cataract, and now, after a successful operation, can see quite well. They are so pleased and grateful as they read line after line on the test-cards after such a long time of blindness. Some there are who have got so used to being blind that it is difficult to get them to use the sight that they have regained. These require a lot of coaxing and a lot of patience, and I doubt if they ever really appreciate what has been done for them. However, these are the few exceptions, and are generally old people who have not seen for many years.

Saddest of all cases are those who are found to be suffering from optic atrophy. They come in complaining that their sight is gradually getting worse and worse, and it is just dreadful to witness their grief when the doctor tells them that there is absolutely no hope for them. It so often happens, too, that it is the breadwinner of the family, and they beg for "some very strong spectacles to help them," vainly hoping that it can't be quite true.

I have heard it said that a nurse's life hardens her and makes her callous to suffering, but my experience is that it widens her sympathy immensely and teaches her boundless patience.

### Everybody's Opinion.

[Correspondence on all subjects is invited, but we cannot in any way be responsible for the opinions expressed by our correspondents. No communication can be entertained if the name and address of the correspondent are not given as a guarantee of good faith, but not necessarily for publication. All correspondents should write on one side of the paper only.]

### THE PENSION FUND.

Annie E. Rossiter writes from the Kent Nursing Institution, Tunbridge Wells: The old proverb says "That the proof of the pudding is in the eating." No doubt many of your readers will remember a discussion on the Royal National Pension Fund for Nurses. Some contended that when a policy was surrendered the policy holder received less than she paid in, etc. Will you allow me to state a fact? In March 1903 I invested a lump sum of £122 in the Pension Fund. One month ago circumstances arose which made it advisable for me to withdraw it. I received a cheque for £127 6s. 11d. I had also to thank the secretary for the kind and courteous manner in which my wishes were met. I shall be grateful if you will find space for this, as after hearing disparaging remarks on the Pension Fund, I am pleased to be able to prove that it is possible to be misinformed or mistaken. I may add that I still hold a policy.

### LOYALTY TO THE DOCTOR.

"L.O.S. and C.M.B." writes: In reference to your note headed "Loyalty to the Doctor" in last week's *Nursing Section*, I should like to say I do not think the case mentioned unusual, though very few nurses would allow themselves to tell a doctor he was in the wrong. In my experience of maternity nursing in small towns and villages, two out of three doctors think it unnecessary to wash their hands before examining the patient. I always point out water and soap which are in readiness, but I am often met by the reply, "Oh, thank you, I have vaseline." Surely if midwives are now so strictly ruled and over-seen, doctors need supervision where they hold supreme sway, and care nothing for cleanliness and antiseptics. This is not all. A ruptured perinæum is thought lightly of by such medical men, and I have met several cases which have been so badly torn, that it has been difficult to give an enema or pass suppository. I should be glad to know if my experience is uncommon.

### NURSES AND UNTIDINESS.

"M. J. L." writes: I think, as we find is the case in all professions, that there are nurses and nurses; that is to say, there are some good, some indifferent, some kind and gentle, and therefore real comforts to their patients, and some like iron. I speak from the experience of a patient having gone through two dangerous illnesses, from the last of which I am only now recovering. My object in writing is to call attention to one failing, which seems to be the most common of any among nurses. It is untidiness; a thing which, though not much considered, perhaps, is yet the cause of more trouble and "work," and consequently of more "ructions" in a house than would be imagined by people who had never come across it. Servants as a rule simply dread the advent of a nurse, and there is really no reason why a woman, who is moreover supposed to be trained not only in medical work, such as bandaging, poultice-making, etc., but in extreme cleanliness and neatness, both essential in a hospital, should in a private house spill candle-grease down the stairs, and upset the carbolic powder all over the lavatory carpet; break handles off jugs and generally prove too strong for the glass and china, not to speak of leaving odds and ends about her patient's room – unless the doctor is expected! Possibly these remarks may result in some practical good. I send them with that hope."

### AN ISOLATION HOSPITAL WITHOUT A TRAINED NURSE.

"A Fever Nurse" writes: I should like to know if "R. A. L. W." considers that all the skill that is required in nursing a tracheotomy case is to abstract, clean, and replace the tube. What about the complications that are likely to arise, and which it is so important for the nurse to detect and report quickly? In all the fever hospitals under the Metropolitan Asylums Board the assistant nurses work under the supervision of a trained nurse, but unfortunately, in some of the smaller fever hospitals, the charge nurses have had no definite training, only experience obtained in small unions, with the result that they cannot bring any skill to bear upon their work, and they also do not understand method and discipline, or the importance and value of time. It is a grave injustice for fever hospitals to accept probationers under such conditions, as they find themselves sadly handicapped when they enter a large hospital or infirmary for general training.

"M. C. T." writes: In answer to "R. A. L. W.'s" letter, I do not wish to infer that only trained nurses know how to remove, clean, and replace a tracheotomy tube. There is no difficulty in performing this simple matter, providing one has been shown how. I still adhere to my statement that, although the nurses had been there several years, they did not know how to remove or clean the tube, for the simple reason that they had never had any one to show them the way, the matron having been caretaker and her husband porter. I think I can say with safety that all charge nurses under the Metropolitan Asylums Board are fully-trained nurses, and have been for the last ten years, to my knowledge. As regards the assistant nurses, I agree that they are well trained under the Metropolitan Asylums Board and are capable of nursing any infectious case. "R. A. L. W." must not compare their assistant nurses to the nurses of the Isolation Hospital I allude to. As to the salary I received and the distance I had to go, I presume that is not the business of "R. A. L. W." My place was not filled by a trained nurse, but by the assistant nurse I mentioned.

### Appointments.

No charge is made for announcements under this head, and we are always glad to receive and publish appointments. The information, to insure accuracy, should be sent from the nurses themselves, and we cannot undertake to correct official announcements which may happen to be inaccurate. It is essential that in all cases the school of training should be given.]

Bury Dispensary Hospital.—Miss Mary W. Wayne has been appointed matron. She was trained at the London Hospital, and has since been sister at the Taunton County Hospital, and at the North Staffordshire Infirmary and Eye Hospital, Stoke-upon-Trent, also assistant matron at the Salford Union Infirmary, Manchester.

Cheadle Union Infirmary.—Miss H. S. Marfleet has been appointed superintendent nurse. She was trained at the Stoke-upon-Trent Workhouse Hospital, where she afterwards became sister.

Corbett General Hospital, Stourbridge.—Miss E. A. Ellett has been appointed staff nurse. She was trained at the General Infirmary, Macclesfield, and at Allt yr yn Hospital, Newport, Monmouthshire.

Croydon Union Infirmary.—Miss Kate M. Goldsmith has been appointed sister. She was trained at Stapleton Infirmary, Bristol, and has since been staff nurse at Camberwell Infirmary. She holds the certificate of the Central Midwives Board.

County Hospital, Shotts, Lanarkshire.—Miss Marion Robertson has been appointed matron. She was trained at the Middle Ward Hospital, Motherwell, and at Crumpsall Infirmary, Manchester where she has since been sister, temporary night-superintendent and theatre-sister. She holds the certificate of the Central Midwives Board.

Devizes Hospital.—Miss M. Sargent has been appointed matron. She was trained at the Royal Devon and Exeter Hospital, where she has since been assistant matron.

East London Hospital for Children, Shadwell, London, E.—Miss Jessie Shaw has been appointed night sister. She was trained at West Ham Hospital, and has since been staff nurse at Pendlebury Hospital, sister of the children's ward at Stockport Hospital, and temporary night sister at Southampton Hospital.

Fleet Cottage Hospital.—Miss Gwenllian Mary Moseley has been appointed matron. She was trained at the Wolverhampton and South Staffordshire General Hospital. She has since been nurse in charge of Barry District Nurses' Home, holiday matron at Bridgend Hospital and at Ebbw Vale Accident Hospital and matron of the Grace Swan Memorial Hospital, Spilsby, Lincolnshire. She has also done nursing in Cromer and in Germany.

Grimsby and District Hospital.—Miss Dora Richardson has been appointed staff nurse. She was trained at the Clayton Hospital, Wakefield, where she was afterwards staff nurse, and undertook sisters' holiday duty. She has also been staff nurse at the Southern Hospital for Women and Children, Manchester.

Midland Counties Home for Incurables, Leamington.—Miss M. O. Cumming has been appointed sister. She was trained at the Liverpool Hahnemann Hospital and has been in charge of the theatre at the Children's Infirmary, Liverpool, charge nurse at the City Hospital, Liverpool, and charge nurse at the City of London Infirmary, Bow.

Mount Vernon Hospital for Consumption, Hampstead.—Miss Hope Ranson and Miss Bertha McDougall have been appointed sisters. Miss Ranson was trained at St. George's Hospital, Bombay, and has had considerable experience in sister work and private nursing. Miss McDougall was trained at the London Temperance Hospital, where she was afterwards staff nurse. She has since been sister at the Manchester Hospital for Women, and sister at the Skin Hospital, Manchester.

Stamford and Rutland General Infirmary.—Miss C. A. M. Coates has been appointed sister. She was trained at Addenbrooke's Hospital, Cambridge, where she was afterwards temporary night sister. She has also been nurse at Buchanan Cottage Hospital, St. Leonards.

Willesden Workhouse Infirmary.—Miss Louisa Amelia Castleman has been appointed charge nurse, and Miss Florence Louise Anne Ewbank has been appointed midwifery nurse. Miss Castleman was trained at the Salford Royal Hospital, and has since been charge nurse at the Eccleshall Infirmary, Sheffield, night nurse at Wolverhampton Workhouse Infirmary, and charge nurse at Dartford Workhouse Infirmary. Miss Ewbank was trained at St. Olave's Infirmary, London, where she has since been charge nurse.

Workhouse Infirmary, Gravelly Hill, Birmingham.—Miss Mary Ann Taylor and Miss Effie Hatfield have been appointed charge nurses. Miss Taylor was trained at Bristol Union Workhouse Infirmary, and has since been charge nurse at the Wakefield Workhouse Infirmary and at the Swansea Workhouse Infirmary. Miss Hatfield was trained at the Bradford Infirmary and has since been sister at the Children's Hospital, Edinburgh.

### Presentations.

Manchester Southern Hospital.—On the occasion of her marriage last Saturday, Miss Davies Brown, matron of the Manchester Southern Hospital, who has been connected with the institution for upwards of 12 years, was the recipient of a handsome purse of gold from the committee, a set of silver brushes, mirror, and travelling trunk from the nursing staff, silver serviette rings and biscuit barrel from the domestic staff, and numerous presents from members of the medical staff.

### Novelties for Nurses.

(By Our Shopping Correspondent.)

### PLASTICINE.

Plasticine is a plastic clay prepared on a new method which retains the plastic character of the substance for almost any length of time. This, of course, is of the utmost importance to the modeller, as the work may be put aside without injury. Another advantage is that the clay can be used without continuously wetting the tools, which is necessary when using ordinary clay. Modelling with Plasticine is quite a hygienic and cleanly occupation, and a most excellent entertainment for small children. Nurses will find it a boon in the sick-room as a means of amusing little patients. It is made in a variety of colours, and can be obtained from all artist colourmen, school supply associations, and the best toyshops, or from the maker, W. Harbutt, Bathampton, Bath. Literature and apparatus appertaining to modelling are sold in connection with Plasticine.

### TRAVEL NOTES AND QUERIES.

By our Travel Correspondent.

Private Nursing on the Riviera (Nil Desperandum).—Since writing to you I have had the opportunity of discussing your plan with residents from both the French and Italian Riviera, and their opinions coincide absolutely with mine—that is, that unless you have sufficient private means to live for a considerable time without work it would be very rash to attempt to establish yourself as a private nurse unattached to any institution. Most English temporary residents, in case of illness, would certainly apply to established institutions, and unless you are well known to many of the doctors you would have no chance. Why risk your little capital? It may be so much needed another day.

Rome for a Month (Nemo).—I am glad to hear from you again. Unless it is necessary do not go to Rome until spring. Dr. Lunn of Endsleigh Gardens has parties arranged for that time on very reasonable terms. All members travel out together, but you can return when you like within the stated dates. Write and ask him for a list of tours about March to May.

Rules in Regard to Correspondence for this Section.—All questioners must use a pseudonym for publication, but the communication must also bear the writer's own name and address as well, which will be regarded as confidential. All such communications to be addressed "Travel Correspondent, 28 Southampton Street, Strand." No charge will be made for inserting and answering questions in the inquiry column, and all will be answered in rotation as space permits. If an answer by letter is required, a stamped and addressed envelope must be enclosed, together with 2s. 6d., which fee will be devoted to the objects of "The Hospital" Convalescent Fund. Ten days must be allowed before an answer can be published.

### Notes and Queries.

**REGULATIONS.**

**The Editor is always willing to answer in this column, without any fee, all reasonable questions, as soon as possible.**

**But the following rules must be carefully observed.**

1. **Every communication must be accompanied by the name and address of the writer.**
2. **The question must always bear upon nursing, directly or indirectly.**

**If an answer is required by letter a fee of half-a-crown must be enclosed with the note containing the inquiry.**

*Epileptic Colony.*

(203) Can you give me any information relating to epileptic colonies? I am much interested in a young man whom I should like to get placed in one if possible.—*Sister Barbara.*

Write to the Secretary of the National Society for the Employment of Epileptics, 12 Buckingham Street, Strand, London, W.C, who will give you full information about the colony at Chalfont St. Peter, Bucks.

*Second Year Nurse.*

(204) Would any hospital take me as second year nurse? I have had a year's probation in a large London general hospital, but left nine months ago, owing to family reasons. I want to take up district work without remuneration, and want to know the way to get sufficient training without starting the three years over again.—*Hungerford.*

Your best plan would be to see if the authorities of the hospital you were in before would receive you for the other two years. You might also write to the superintendent of the Queen Victoria's Jubilee Institute, Victoria Street, S.W., for advice.

*Visiting Nurse.*

(205) There is an association in London I believe where daily visiting nurses are sent out. Can you give me the address?—*A. D.*

The address you require is Marylebone Association, 126 Seymour Place, Marylebone, where you can learn full details about the visiting nurse. With regard to your friends not having yet received answers to their queries you must remember that owing to the enormous number of letters we receive it is impossible to publish replies immediately after the receipt of the question.

*Central Midwives Board.*

(206) I am a trained nurse of several years' experience and practice in maternity and midwifery work, but have never taken the L.O.S. certificate. I should be glad to know if it would be possible to obtain a certificate from the Central Midwives Board free of charge. What is the address of the chief office of the Central Midwives Board?—*Anxious.*

You could not obtain the Central Midwives Board certificate without paying a fee. The offices of the Board, where all information will be given, are at 6 Suffolk Street, Pall Mall, S.W. The Scientific Press, 29 Southampton Street, Strand, publishes a little work "How to Become a Certified Midwife."

*Certificate.*

(207) Would you kindly inform me whether a certificate is any good to a girl who goes into training at 18 in a hospital of 50 beds?—*Matron.*

Such a certificate would be of little value to her in her nursing career.

*Married Women.*

(208) Will you kindly let me know whether any institution trains married women as medical and surgical nurses, and, if so, where? I am left badly off owing to the intemperance of my husband, who is unable to maintain me. I have excellent references from clergymen and doctors. Will you please help me?—*Miriam.*

(209) We are afraid that you will have some difficulty in training unless you go in for maternity work. Your best plan would be to study "How to Become a Nurse. The Nursing Profession, How and Where to Train," published by the Scientific Press. Then write to some matrons and explain your circumstances fully.

**Handbooks for Nurses.**

| | Post Free. |
|---|---|
| "A Handbook for Nurses." (Dr. J. K. Watson.) ... | 5s. 4d. |
| "Nurses' Pronouncing Dictionary of Medical Terms." ... | 2s. 0d. |
| "Art of Massage." (Creighton Hale.) ... ... ... ... | 6s. 0d. |
| "Surgical Bandaging and Dressings." (Johnson Smith.) | 2s. 0d. |
| "Hints on Tropical Fevers." (Sister Pollard.) ... ... | 1s. 8d. |

Of all booksellers or of The Scientific Press, Limited, 28 & 29 Southampton Street, Strand, London, W.C.

### For Reading to the Sick.

"MINISTERING SPIRITS."

"Ministering Spirits" sent
Both to follow and prevent,
Lest the loved ones Thou dost own
Dash their foot against a stone.
But with gentlest, tenderest love
All their other care above,
They with earnest hearts and eyes
Watched the wondrous sacrifice.

And when He on high ascended
Wrapt in clouds, by hosts attended,
Hailed while entering heaven's abode,
Son of man! and Son of God.
Two in robes of white arrayed
Willingly behind Him stayed,
To uplift the hearts that yearn
O'er His loss to His return.

*Dr. J. S. Monsell.*

As a simple matter of fact, angels do make much appearance in the Bible, New Testament as well as Old—they do discharge important duties; they are invested with immense powers for the work they have to do, and there is not one single word even to suggest that, having done their work and served their day, they are now off the scene. So far from this being the case, the immediate and necessary service of angels to the individual believer is much more distinctly stated and seriously impressed in the New Testament than in the Old.—*Rev. T. Mozley.*

The angels of God are ever with us. They haunt us at every turning. Do not be indifferent to their presence! Do not think them absent because you cannot catch the expression of their face, or trace the outlines of their form The spiritual presences are the most real presences. Let not the ministries of life and death, of the visible and invisible world, be lost upon you. In God's good I plead with you to welcome the heavenly messengers.—*Dr. T. Hunter.*

I believe no soul is left to wing its viewless flight to Paradise in solitude. I believe the "Gloria in Excelsis" of the shining host of God welcomes the disembodied spirit upon the confines of the new world. I remember hearing once of a little dying child shrinking timidly from the idea of going alone; but just before the end there came a spirit of sublime confidence, a supernatural opening of vision, a recognition of some companionship, and the little one cried out, "I am not afraid; they are all here." Perhaps they were the angels. Lazarus was carried by the angels into Abraham's bosom. I believe the chamber of the dying is filled with the holy angels; I believe the rustle of their wings is around you as you kneel to offer the commendatory prayer.

*Archdeacon Basil Wilberforce.*

One who looks to helping angels in their work should make this world a school for lovingness. Let him begin by loving "what he has seen"—every living thing that comes in his way—and he will go on to love the angels too; for they also love God's creatures, and treasure up the good and happiness that is brought to light. Thus he may be brought, by easy steps, to love Christ and God, and loving God, to come to know him, which our Lord tells us is eternal life.

*Rev. H. Latham.*

